# A new pycnodont fish, *Scalacurvichthys naishi* gen. et sp. nov., from the Late Cretaceous of Israel

**DOI:** 10.1080/14772019.2017.1330772

**Published:** 2017-06-14

**Authors:** John J. Cawley, Jürgen Kriwet

**Affiliations:** Department of Palaeontology, University of Vienna, Geozentrum, Althanstraße 14, 1090 Vienna, Austria

**Keywords:** Tethys, Cenomanian, Pycnodontomorpha, phylogeny, morphology, taxonomy

## Abstract

A new pycnodont fish from the early–mid Cenomanian, Late Cretaceous, of the ‘Ein Yabrud quarry near the village of Beit Eil in Israel is the first pycnodont fish to be described from this locality. Due to the locality where it was found, *Scalacurvithys naishi* gen. et sp. nov. is considered an inhabitant of reefal waters interspersed with lagoons in the eastern Tethys Sea. *Scalacurvichthys naishi* is notable for its protruding, hook-shaped first dorsal ridge scale above a large triangular dermatocranium, a deeply sloped and antero-posteriorly shortened skull and bifurcated cloacal scales. The bifurcating scales are a new character previously unknown in pycnodontomorph fishes but have been discovered in two more taxa, which indicates a new type of character that will be useful for future phylogenetic analyses of pycnodontomorph fishes. The new taxon is a member of Pycnodontidae and we conducted a phylogenetic analysis to establish its relationships to other pycnodont fishes. Our results reveal that *Scalacurvichthys naishi* is a well-resolved member of the subfamily Pycnodontinae.

http://zoobank.org/urn:lsid:zoobank.org:pub:04383E2A-551A-4F57-8996-68E06EFA52E0

## Introduction

Pycnodontomorpha is a superorder of actinopterygian fishes that contains the orders Pycnodontiformes and Gyrodontiformes (Nursall 2010). These fishes were a significant component of marine, freshwater and brackish ecosystems (e.g. Longbottom 1984; Poyato-Ariza *et al*. 1998) from the Late Triassic to the Eocene (Tintori 1981; Longbottom 1984). The typical bauplan for these fishes is a deep, laterally compressed body. Molariform teeth are usually present on the prearticulars and vomer, while small grasping or incisiform teeth are present on the dentary and premaxilla (Nursall 1996a). The maxillae are endentulous. This kind of dentition is considered characteristic for pycnodonts (e.g. Kriwet 2001a; Poyato-Ariza & Wenz 2002; Nursall 2010). Many taxa were most likely durophagous, consuming heavily armoured invertebrates such as crustaceans and gastropods. However, extensive diversities of premaxilla/dentary tooth morphologies and varying arrangement, shape and thickness of the molariform teeth mean that it is likely that pycnodontomorphs as a whole might have filled a wide variety of ecological niches (Poyato-Ariza 2005). The height of their morphological disparity, particularly for Pycnodontiformes, was during the Cenomanian (Marramà *et al*. 2016), and included members of families such as Gladiopycnodontidae (Taverne & Capasso 2013, 2014a), Gebrayelichthyidae (Nursall & Capasso 2004; Taverne & Capasso 2014b), Trewavasiidae (Gayet 1984; Nursall & Capasso 2008) and Coccodontidae (Kriwet 2004; Capasso *et al*. 2010; Taverne & Capasso 2014c). Most of these Cenomanian pycnodont fishes are known from the Middle East, mainly from the famous fossil vertebrate localities of Hajoula, Haqel and Namoura in Lebanon (e.g. Davis 1887; Hückel 1970; Forey *et al*. 2003). Hussakof (1916) reported an additional Late Cretaceous pycnodont fish record representing an isolated prearticular dentition from the region of Beirut, which he assigned to *Coelodus*. Other Late Cretaceous Middle East pycnodont records are from the Maastrichtian of Syria (isolated teeth and jaw fragments; Bardet *et al*. 2000) and the Late Cretaceous (Cenomanian?) of Iran identified as *Coelodus* by Priem (1908).

Pycnodontomorph fish remains from Israel, conversely, have been considered only sporadically up to now. Haas (1979) indicated the presence of a pycnodont dentition as stomach contents of the basal snake *Pachyrhachis* from the Cenomanian of Israel, and Lewy *et al*. (1992) indicated the presence of two endocranial casts in the Campanian Mishasha Formation, which they identified as belonging to *Micropycnodon*. However, no detailed descriptions or figures of these remains were provided, so currently their taxonomic affinities remain ambiguous. The new genus described here is the first taxonomically identified pycnodont genus from the Late Cretaceous of Israel. It is represented by a single, mostly complete specimen that allows analysing its systematic position within Pycnodontiformes using cranial and postcranial characters. The goals of this study, consequently, are to provide a detailed morphological account of this new taxon and to establish its interrelationships within pycnodontomorph fishes, employing rigorous cladistic principles.

## Locality and geological setting

The single specimen is preserved in a fine-grained and yellowish limestone slab, which is very thin, indicating that it comes from a very finely laminated limestone deposit. The specimen was obtained as part of a larger collection of fossil fishes from Israel by the State Museum of Natural History Karlsruhe (Germany) in the early 1980s and, unfortunately, no detailed records are available. The label states that the specimen is from the Late Cretaceous (*c*. 100 Ma) of Bethel in the Negev Desert, Israel. To our knowledge, however, no fossiliferous and finely laminated limestones occur near the village of Bethlem (assuming that the label may refer to this village), which is located south of Jerusalem. Conversely, a detailed comparison by us of the lithological features of the limestone slab containing the specimen revealed that it is identical to those reported from the famous fossil fish locality of ‘Ein Yabrud in the Judean Hills. Lithologically similar fossil-bearing limestone layers also occur in the western vicinity of Jerusalem (Giv'at Sha'ul), including completely articulated fossil fishes (Raab & Chalifa 1987). These layers are supposed to be of late Cenomanian age and thus are slightly younger than those from ‘Ein Yabrud (see below). The major difference between the two localities is expressed in the preservation of vertebrate fossils due to different taphonomic regimes. According to Gayet (1986), fossil vertebrates from ‘Ein Yabrud display variable post-mortem fusion of cranial bones, with the post-cranial skeleton being well preserved. The cranial skeleton in fossil fishes from the locality of Giv'at Sha'ul, conversely, additionally is well preserved, with single bones being easily distinguishable. The specimen described here displays fused cranial but well-identifiable postcranial elements, and it therefore is most parsimonious to suppose that this specimen comes from one of the many ‘Ein Yabrud quarries.

The main fossil fish locality is near the village of ‘Ein Yabrud, which is located *c*. 20 km north of Jerusalem and *c*. 7 km north-east of Ramallah. Here, thin limestone flags were quarried for building stone and facing, yielding a spectacular Late Cretaceous fauna and flora (e.g. Rieppel *et al*. 2003). The fossil-bearing horizon, which comprises only a short sequence (*c*. 2.5–3.5 m maximum) of finely laminated and yellowish to reddish exposed limestone layers, is geographically restricted to a rather small area north of Ramallah (e.g. Chalifa 1985). The age of the fossiliferous beds has been controversially discussed for many years and they were variously placed either into the early Cenomanian Bet Meir Formation or the late Cenomanian Amminadav Formation (e.g. Braun 1970; Lewy & Raab 1976; Chalifa & Tchernov 1982; Chalifa 1985). The fossil-bearing strata are now accepted to be early–middle Cenomanian in age (Tchernov *et al*. 2000; Rieppel *et al*. 2003; Head 2015). Unfortunately, no detailed information about the precise stratigraphical level of individual fossils within the ‘Ein Yabrud quarries is available and we therefore assume an early–middle Cenomanian age for all fossil fishes from these quarries, in agreement with previous publications (see above). The depositional area of the finely laminated limestone layers is interpreted as a quiet lagoon in a reefal region (Scanlon *et al*. 1999).

Quarries consisting of finely laminated limestones of various colours and containing fossil fishes similar to those of the ‘Ein Yabrud quarries occur near the villages of Baytin and Beit El, which are located *c*. 4 km south-west of the village of ‘Ein Yabrud (e.g. Braun 1970). We assume that the name ‘Bethel’ given on the specimen label is a misspelling of Beit El and that this, therefore, represents the type locality of the new taxon.

## Material and methods

The single specimen consists of an almost completely and fully articulated specimen, lacking, however, some cranial and postcranial details due to taphonomic processes. Several morphological elements especially of the postcranial skeleton are preserved and recognizable only as imprints. Therefore, a cast of the caudal fin was prepared to better analyse its morphology. Some areas of the specimen such as the opercular skeleton were heavily overlain by rocky matrix, and to get a more accurate description of its morphology preparatory work was undertaken. Rocky matrix was removed with a Veit pen drill. The specimen was mechanically prepared under a Mantis Elite eyepiece-less microscope at 8 × and 10 × magnification while removing this excess rocky matrix. After preparation, the specimen was photographed using a Nikon® D7000 camera with a 60 mm Micro Nikkor lens that is stabilized by a wall-mounted camera stand. The aperture was set at F22 and the shutter speed used was 0.50 seconds. Drawings were made using camera lucida.

The nomenclature used by many authors for describing dermal skull bones in actinopterygians follows the traditional (‘orthodox’) terminology for mammals and is not based on homology criteria. The difficulties in establishing homology and the great variability of dermal elements in the heads of actinopterygian fishes (see, for instance, Gregory 1933) led to the publication of different names for the same bone, that are rather unintelligible (see also Schultze & Arsenault 1985). Therefore, the terminology for the dermal head skeleton used in this study follows that of Jollie (1962) and Schultze (1993). The terminology for the caudal skeleton follows Arratia & Schultze (1992) as well as Schultze & Arratia (1986). The terminology for the squamation follows Poyato-Ariza & Wenz (2002, 2005).

The term ‘pycnodontomorph’ refers to any member of Pycnodontomorpha, and the term ‘pycnodontiform’ to Pycnodontiformes. ‘Pycnodontid’ identifies members of the family Pycnodontidae, and ‘pycnodontin’ signifies members of the sub-family Pycndontinae.

The phylogenetic relationships of the new pycnodont fish from Israel were explored using cladistic principles. The analyses in this study were conducted using Winclada software, version 1.00.08, of Nixon (1999, 2000). The character database used for this analysis was that of Poyato-Ariza & Wenz (2002), with the family Coccodontidae removed due to the revision of taxa previously considered members of Coccodontidae now being placed in the family Gladiopycnodontidae (Taverne & Capasso 2013, 2014a, b, 2015), a family with no phylogenetic work carried out on it, and Trewavasiidae (Nursall & Capasso 2008). This makes Coccodontidae a polypheletic group, and as such creates noise in the data when a new taxon such as *Scalacurvichthys* is added to the original database of Poyato-Ariza & Wenz (2002). Phylogenetic work on a separate family or families of pycnodont fishes is an undertaking outside the scope of this paper and will be presented elsewhere with a revised data matrix for pycnodontiform fishes. Due to recent taxonomic studies, what was the genus *Macromesodon* in the Poyato-Ariza & Wenz (2002) phylogenetic analysis is now classified as *Turbomesodon* (Poyato-Ariza & Wenz 2004), with *Macromesodon macropterus* now being *Turbomesodon relegans*, *M. bernissartensis* now being *T. bernissartensis*, and *Macromesodon* cf. *M. bernissartensis* now being *T. praeclarus*. *Apomesodon surgens* is now assigned to *Macromesodon* (Poyato-Ariza & Wenz 2004), while *Apomesodon gibbosus* is more problematic as Poyato-Ariza & Wenz (2004) noticed that different specimens of this taxa show two different morphologies, leading them to classify them into two species of *Macromesodon*, *M. macropterus* and *M. gibbosus*. This makes it difficult to decide which specimens of *Apomesodon gibbosus* were included in the phylogenetic analysis of Poyato-Ariza & Wenz (2002). However, one character (Character 70: number of anal axonosts) and the corresponding character state 0 (10–19) has helped us reveal that the specimen which was used in the analysis is now *Macromesodon macropterus*. This is because a diagnostic character of *M. macropterus* is the number of anal axonosts, which is fewer than 20 (Poyato-Ariza & Wenz 2004). So *Apomesodon gibbosus* is renamed *Macromesodon macropterus* in this phylogenetic analysis. The final taxonomic change that we put in this analysis was that of *Nursallia gutturosum* now being called *Paranursallia gutturosa* (Taverne *et al*. 2015).

In our phylogenetic analysis, all characters were treated as unordered and unweighted, conversely to the original data analysis. We used unordered characters because ordering characters assumes a priori interpretations of evolutionary events onto a phylogeny. The following settings were employed: heuristic search, multiple TBR + TBR algorithm that searches for trees using a tree bisection-recombination method of branch swapping with 1000 replications, DELTRAN optimization that puts changes on the tree as late as possible, and initial MaxTrees setting of 30,000. The bootstrap option with 1000 replications was used to calculate the support of nodes.

## Institutional abbreviations

**SMNK**: State Museum of Natural History Karlsruhe, Germany; **JME**: Jura Museum Eichstätt, Germany; **MNHN**: Muséum national d'Histoire naturelle, Paris, France.

## Systematic palaeontology

Class **Osteichthyes** Huxley, 1880

Subclass **Actinopterygii** Cope, 1887

Series **Neopterygii** Regan, 1923

Division **Halecostomi** Regan, 1923 *sensu* Patterson, 1973

Order **Pycnodontiformes** Berg, 1937

Family **Pycnodontidae** *sensu* Nursall, 1996a

cf. Subfamily **Pycnodontinae** Poyato-Ariza & Wenz, 2002

Genus ***Scalacurvichthys*** gen. nov.

### 

#### Type species

*Scalacurvichthys naishi* sp. nov.

#### Age

Early–middle Cenomanian, Late Cretaceous.

#### Diagnosis

Pycnodontid fish with the following autapomorphic characters: large triangular dermocranium; large anteriorly curved first dorsal ridge scale which protrudes above the skull roof; 11 dorsal axonosts; single post-cloacal ventral ridge scale; position of anal fin (preanal length/standard length) being at 70–79%; large, anterior and posterior bifurcating cloacal scales. Unique combination of plesiomorphic and derived characters: body outline intermediate between discoid and fusiform; body height 50% of standard length (SL); dermocranial fenestra absent; premaxillary bone with two teeth and no olfactory fenestra; 21 neural vertebrae excluding the caudal peduncle; 30–31 caudal fin rays; four epurals and 10 hypochordals in the caudal endoskeleton; hypochordals six, seven and eight seem to be fused into a large fan-shaped ossification.

#### Derivation of name

The genus name is derived from the Latin noun ‘*scala*’ meaning ‘scale’, the Latin adjective ‘*curva*’ meaning ‘curved’ in allusion to the raised, anterior-facing first dorsal ridge scale protruding above the skull roof, characteristic of this genus, and the Greek noun ‘ἰχθύς’ meaning ‘fish’.

***Scalacurvichthys naishi*** sp. nov.

(Figs 1–5)

**Figure 1.**
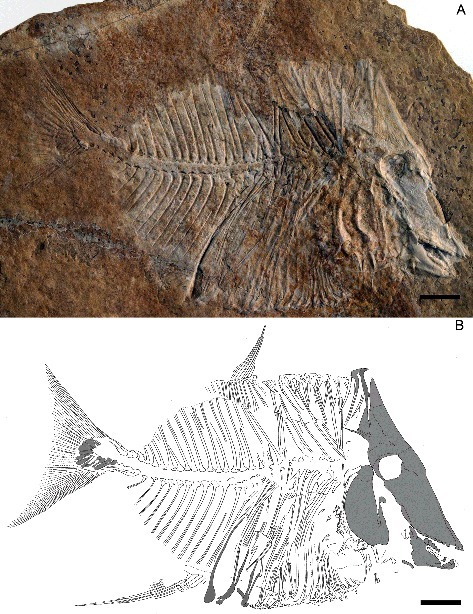
**A**, *Scalacurvichthys naishi* gen. et sp. nov., holotype (SMNK-PAL. 8613). **B**, camera lucida drawing of *Scalacurvichthys naishi* gen. et sp. nov.; dashed lines indicate the restoration of incompletely preserved structures; bones shaded in grey are reconstructions while the rest of the drawing is the original specimen. Scale bars = 1 cm.

**Figure 2.**
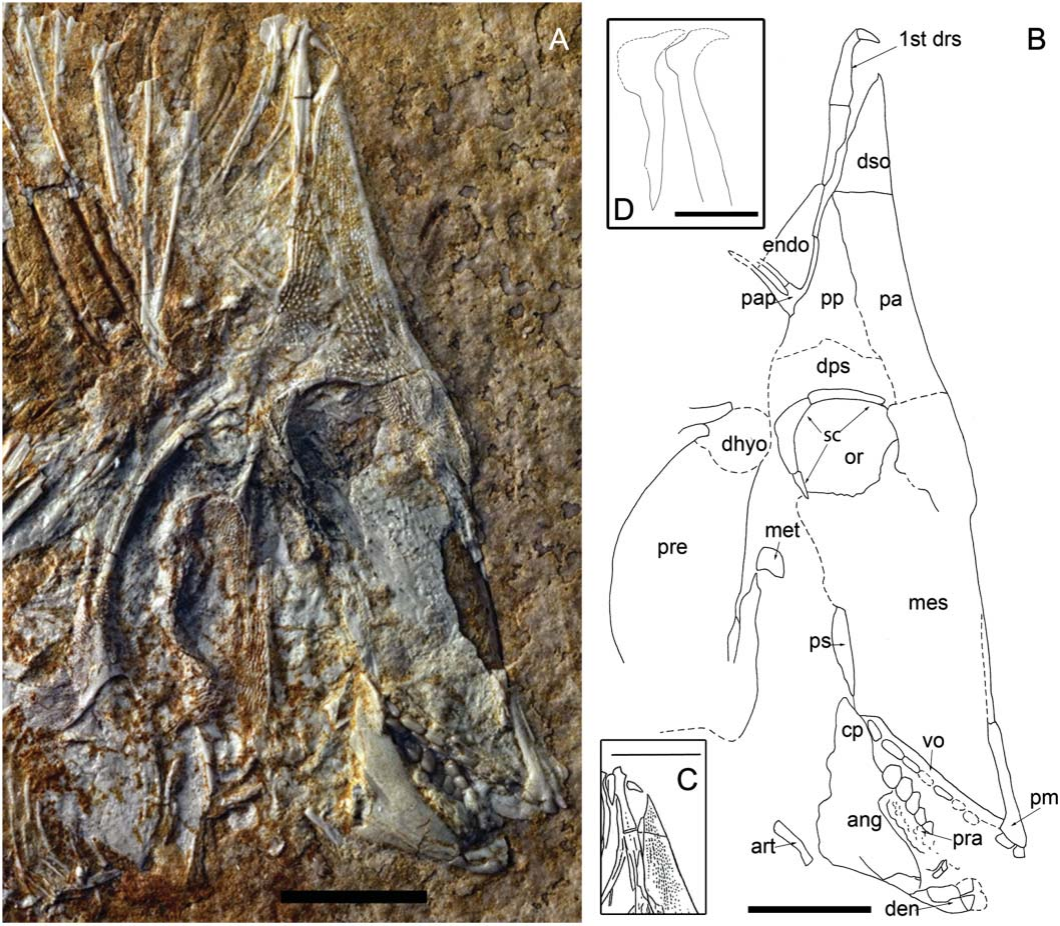
**A**, skull of *Scalacurvichthys naishi* gen. et sp. nov., holotype (SMNK-PAL. 8613) with forward-facing first dorsal ridge scale. **B**, camera lucida drawing showing restored position of scale tip in life. **C**, camera lucida drawing of first dorsal ridge scale as it is seen in the holotype showing original position of the tip of the spine. **D**, restoration of first two dorsal ridge scales revealing probable morphology; dashed lines indicate the restoration of incompletely preserved structures. Abbreviations: 1st drs, 1st dorsal ridge scale; ang, angular bone; art, articular bone; cp, coronoid process; den, dentalosplenial; dhyo, dermohyomandibular; dps, dermopterosphenotic; dso, dermosupraoccipital; endo, posteriorly exposed endocranium; mes, mesethmoid; met, metapterygoid; or, orbit; pa, parietal; pap, post-parietal process; pm, premaxilla; pp, post-parietal bone; pra, prearticular bone; pre, preoperculum; ps, parasphenoid process; sc, sclerotic ring; vo, vomer. Scale bars: A–C = 1 cm; D = 50 mm.

**Figure 3.**
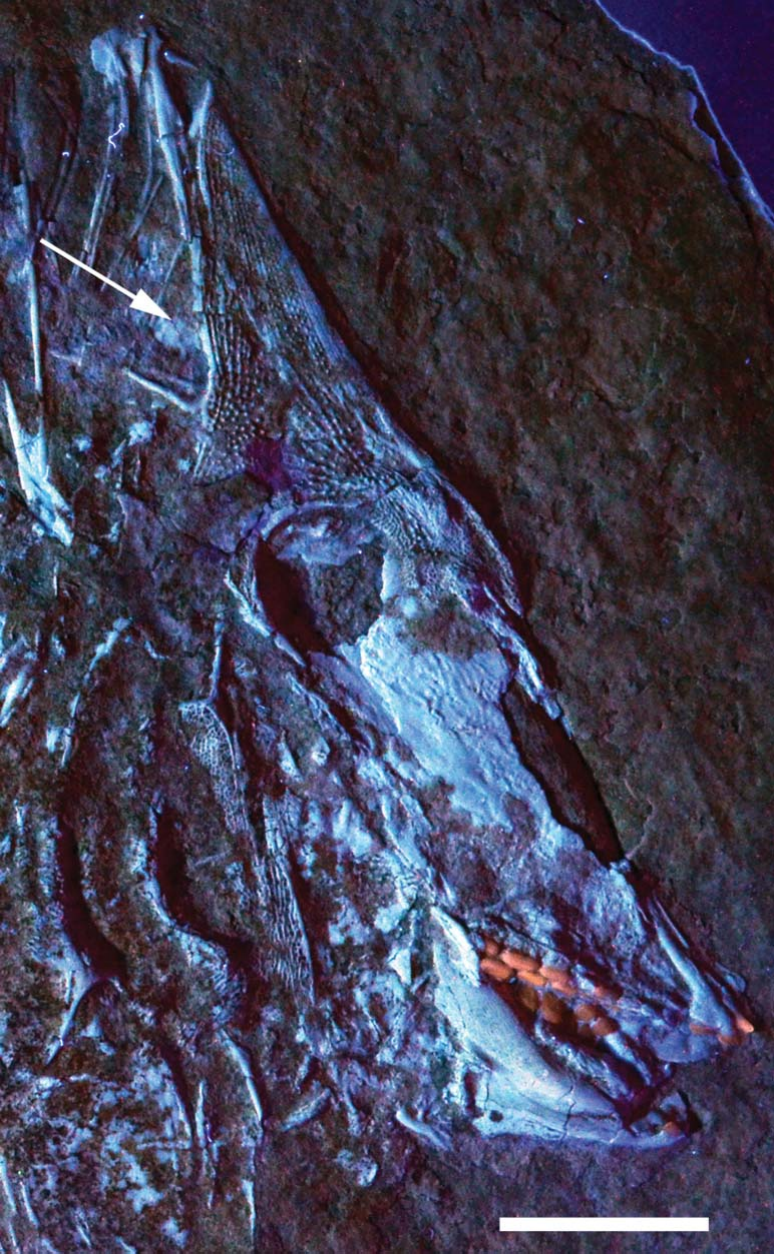
Skull of *Scalacurvichthys naishi* gen. et sp. nov., holotype (SMNK-PAL. 8613) under UV light in order to show the preserved remains of the posterior exposed endocranium to which the arrow points. Scale bar = 1cm.

**Figure 4.**
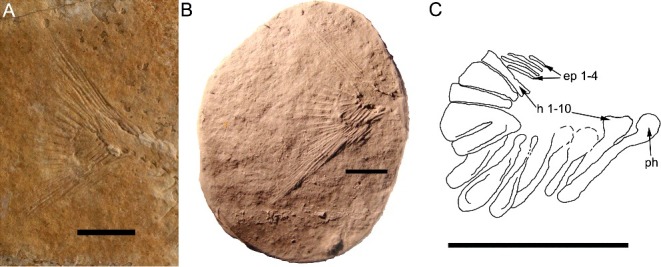
**A**, imprint of caudal fin of *Scalacurvichthys naishi* gen. et sp. nov. holotype (SMNK-PAL. 8613). **B**, cast of caudal fin, mirrored; anterior to the right. **C**, camera lucida drawing based on cast of caudal fin; dashed lines indicate the restoration of incompletely preserved structures. Abbreviations: ep 1–4, epichordals 1–4; h 1–10, hypochordals 1–10; ph, parhypural. Scale bars = 1 cm.

**Figure 5.**
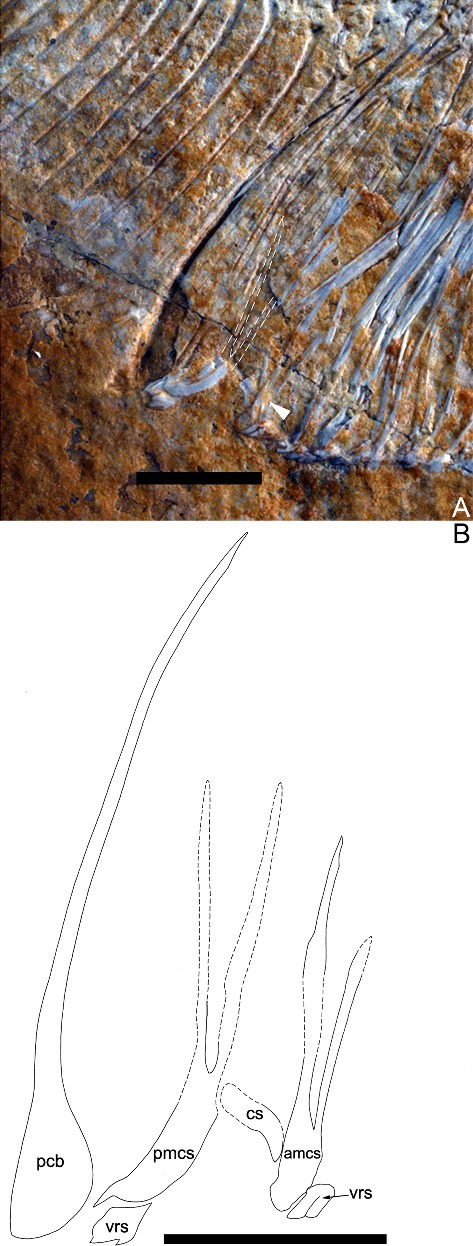
**A**, cloaca of *Scalacurvichthys naishi* gen. et sp. nov. holotype (SMNK-PAL. 8613); dashed white lines indicate the restoration of incompletely preserved structures; tip of right branch of posterior modified cloacal scale overlain by disarticulated flank scales therefore not shown. **B**, camera lucida drawing; probable shape of posterior modified cloacal scale is reconstructed using dashed lines. Abbreviations: amcs, anterior modified cloacal scale; cs, cloacal scale; pcb, postcoelomic bone; pmcs, posterior modified cloacal scale; vrs, ventral ridge scale. Scale bars = 1 cm.

#### Type specimen

Holotype SMNK-PAL. 8613. Complete specimen.

#### Age

Bet Meir or the slightly younger Amminadava Formation, middle part of the Judea Group, early to middle Cenomanian, early Late Cretaceous.

#### Type locality

Limestone quarry near the village of Beit El, Binyamin Region, West Bank, Israel.

#### Geographical distribution

Only known from the type locality.

#### Stratigraphical range

Early–middle Cenomanian, Late Cretaceous.

#### Diagnosis

As for the genus. Only known species.

#### Derivation of name

The name of the new species is dedicated to Dr Darren Naish who is currently writing a book on the entire vertebrate fossil record and is prolific in publishing research on dinosaurs, pterosaurs and marine reptiles amongst many other groups of tetrapods.

#### Description

##### Size and general morphology

This new taxon is a small-sized pycnodont (Fig. 1). The SL of the new taxon is 12.3 cm. Its general body shape is oval with a deeply sloped skull. The snout is very deeply turned down. At a maximum body height of 6 cm it is relatively high bodied but distinctly less high than other pycnodonts such as *Gyrodus* from the Late Jurassic. The highest point of the body is at the first dorsal ridge scale which would have protruded 6 mm above the dermosupraoccipital when the animal was still alive. This dorsal ridge scale is spine-like and elongate, and curves anteriorly at the tip.

The dermosupraoccipital is triangular in form and is directly dorsal to the parietal bone, the only bone in the skull roof that it contacts. The dermocranial fenestra is absent from this specimen. The ventral apex appears to be at the posterior edge of the post-coelomic bone just before the anal fin. Conversely, the dorsal apex appears to be absent. The flank scales are reduced to scale bars, with dorsal flank scales covering only the abdomen while ventral flank scales cover both the abdomen and trunk. Scales are absent from the body posterior to the post-coelomic bone. The cleithrum appears to be a large bone that encircles the opercular skeleton, but the bone mixing with the rocky matrix makes its true form difficult to discern. Other than three fragmented hypochordals in the caudal fin and neural spines in the caudal peduncle, all of the body posterior to the post-coelomic bone is preserved as an imprint in the rock only. The dorsal and anal fins are extremely sickle shaped with no evidence of either fin contacting the caudal fin. The body tapers posteriorly to the rather thin and un-keeled caudal peduncle. The caudal fin has a height of 4 cm and has a convex margin at the distal border. There is no evidence of pectoral or pelvic fin preservation in this specimen.

##### Skull roof

The boundary between cranial roof bones is difficult to discern due to taphonomic processes that are characteristic for fossil fishes from ‘Ein Yabrud. Additionally, several bones seemingly were lost post-mortem.

The parietal (Figs 1, 2) is a large wedge-shaped bone ventral to the dermosupraoccipital and anterior to the post-parietal and dermopterosphenotic. The dorsal margin of the parietal is flat. A faint suture in front of the orbit seems to indicate the ventral margin of the parietal, which is dorsal to the mesethmoid. The posterior margin of the bone slopes to the right in an antero-ventral direction and expands posteriorly over the dorsal margin of the orbit. This bone is heavily ornamented with tubercles. Tubercles on the anterior border are highly organized into tightly packed rows, in contrast to the widely spaced tubercles found elsewhere on the bone. On the ventral portion of the parietal where tubercles are absent, the surface is dotted with pits.

The post-parietal (Figs 1, 2) is situated ventral to the first dorsal ridge scale and posterior to the parietal bone. The suture between the parietal and the post-parietal is uneven. Tubercles are present all over the bone, which form into ridges at the postero-dorsal border of the post-parietal. A parietal process protrudes from its posterior border. Two long, thin branches jut out facing in a posterior direction. The distal ends of the branches are obscured by rocky matrix, which may also obscure additional branches of the parietal process.

The ventral boundary of the post-parietal is hard to observe but is assumed to occur where the bony surface becomes smoother. We interpret this bone to be the dermopterosphenotic due to the change in ornamentation and its location, being ventral to the post-parietal and dorsal to the posterior margins of the orbit.

The dermosupraoccipital (Figs 1, 2) is a tall and broadly triangular bone placed on top of the skull roof. It is dorsal to the parietal. The tip of the bone is pointed and has a slight convex expansion on the right side and concave margin on the left. Tubercles cover the anterior portion of the dermosupraoccipital. Posterior to the dermosupraoccipital is the first dorsal ridge scale which is incorporated into the skull roof as seen in all known pycnodonts (Nursall 1996b; Poyato-Ariza & Wenz 2002) with the exception of Gladiopycnodontidae (Taverne & Capasso 2013).

#### Orbit and neurocranium

Bones surrounding the orbit are fragmented and only the dorsal and posterior portions are preserved. The dorsal sclerotic ring fragments have rounded anterior margins and flat dorsal margins. Sclerotic rings widen in a posterior direction. The posterior sclerotic ring is crescent shaped and dotted with pits on its surface. The antero-ventral corner of the posterior sclerotic ring ends in a distal point, and has wavy posterior margins and flat anterior margins.

Inside the orbit, an elongate, curved bone with rounded distal ends is present in the ventral half of the orbit (Figs 1, 2). In the postero-dorsal corner of the orbit, rocky matrix obscures the remaining portions of this bone. The bone widens near the rocky matrix. The shape and position of the bone indicates a posterior orbital bone from the left-hand side of the fish.

In the dorsal half, a large bone gently curves down in an antero-ventral direction to a point. From the point, the bone has a concave margin until the fragmentation obscures the true nature of the margin. The identity of the bone is unknown but it may possibly represent the orbitosphenoid. This bone is not heavily ossified and has numerous fractures along its ventral margin but is otherwise unornamented. The bone overlying the probable orbitosphenoid has a concave posterior margin and a lateral medial bony process slightly raised from the surrounding bone. The anterior margin of the bone is convex. The bone is heavily intermixed with rocky matrix so its identification is difficult. No infraorbitals are preserved.

A smooth, unornamented bone appears to be overlain by the limbs of the post-parietal process. Due to the anteriorly extreme shortening of the skull roof in this specimen, it is probable that this could be the posterior margin of the endocranium. However, due to the intermixing of this probable endocranial portion with the rocky matrix, it is difficult to identify the shape and true identity of this bone. Doing preparatory work to remove this sediment could be problematic due to the fact that the post-parietal process limbs overlie this bone and there is a high chance they could be damaged. The presence/absence of a postcephalic lacuna thus cannot be confirmed. Exposing the specimen to UV light, however, reveals smooth bony remains dorsal to the post-parietal process and posterior to the dermatocranium (Fig. 3, arrow). The morphology and position of these remains correlate well with the posteriorly exposed endocranium in pycnodontids.

#### Jaw bones and dentition

The premaxilla (Figs 1, 2) has a long, thin process protruding dorsally from the tooth-bearing portion. The true length of this process is unclear due to fragmentation in front of the skull. Two incisiform teeth are present and have a flat base with a pointed anterior margin and rounded posterior margin. The maxilla is not preserved in this specimen.

The dentalosplenial (Figs 1, 2) is a small jaw bone with a broad anterior margin that narrows in an antero-posterior direction. Two teeth are present on the dentalosplenial. The incisiform teeth have a flat base with a rounded posterior margin. The posterior margin is expanded in the first tooth. On the second tooth, the anterior margin narrows in a dorso-ventral direction.

Two rows of teeth are preserved in the vomer in the roof of the mouth (Figs 1, 2). The left lateral row shows six teeth preserved. There is a hollow between the second and fourth tooth, which indicates a tooth was present there while the fish was alive. This brings the total of teeth in the left lateral row to seven. The shape of these teeth is an elongate oval with a smooth surface, increasing in size in an antero-posterior direction. The median row has six teeth preserved. Teeth start out as elongate ovals but become much broader the farther back in the jaw they go. A shallow hollow is present medially in all median-row teeth with the exception of the first one, which is fully flattened. Crenulated ridges are only present around the outer margin of the final tooth of the main middle row; all other teeth are smooth.

There are three rows of teeth present in the prearticular bone (Figs 1, 2). The right lateral row has four teeth preserved, which are globular with a rounded contour and smooth surface. Seven teeth are preserved in the principal median row. Fully preserved teeth are elongate with an oval contour and smooth surface. Rocky matrix has formed over parts of the teeth as well as uplifted sections of the right lateral row. There are only three teeth preserved on the left lateral row, which are mostly embedded in rocky matrix. Teeth that are preserved show a smooth, flattened, elongate oval shape similar to those of the principal row.

The angular is a large bone, which makes up the bulk of the lower jaw (Figs 1, 2). It is positioned posterior to the dentalosplenial, ventral to the prearticular bone and anterior to the articular bone. The coronoid process has a rounded margin, which is posterior to the vomer. Ridges are present in the postero-dorsal region of the angular.

The articular bone is a small, thin and elongate bone located at the postero-ventral corner of the angular (Figs 1, 2). The dorsal portion of the articular has a rounded margin and faces in a cephalo-caudal direction. The ventral margin is flat and located closer to the ventral border of the angular. The articular is broad at the ventral margin and narrows in a cephalo-caudal direction. It is a smooth bone without ornamentations.

The mesethmoid is a large bone, but mostly preserved only as imprint so that possible ornamentation patterns remain unknown (Figs 1, 2). This bone seemingly takes up most of the skull. It is ventral to the orbit and parietal and dorsal to the vomer. The posterior border is ventral to the posterior portion of the orbit and dorsal to the coronoid process of the angular.

The parasphenoid is overlain by the mesethmoid and can be observed only partially at the postero-ventral corner of the mesethmoid. The observed portion of this bone has a posterior convex margin and a smooth surface.

The bones of the palatoquadrate arcade are incompletely preserved. The metapterygoid is a small bone with a flat base, convex anterior margins and a flat posterior margin. It has a smooth surface with no ornamentation. This bone is located postero-ventral to the orbit and posterior to the mesethmoid.

##### Opercular series

Much of the opercular skeleton is heavily crushed and sutures cannot be seen so the extent of the bone size and its shape remain unknown at present.

Even though much of the opercular skeleton is fragmented or eroded, we interpret its structure thus: the preoperculum is a large, deep, broad bone taking up much of the antero-ventral portion of the opercular skeleton. It is located postero-dorsal to the metapterygoid (Figs 1, 2) and dorsal to the singularly preserved branchiostegal ray. The preoperculum is heavily ornamented with ridges occurring in a criss-cross pattern.

A large process protrudes *c*. 4 mm antero-dorsally from the preoperculum and is also ornamented with ridges. The distal margin of the process is flat and a posterior lateral process protrudes from the distal tip. This process has a flat dorsal margin and mixes with rocky matrix in its ventral region so the shape of the ventral margin remains unknown. The process is found postero-ventral to the posterior plate of the orbit.

Between the dorsal preopercular process and the antero-dorsal extension of the cleithrum lies the dermo-hyomandibular. The dermohyomandibular is located postero-dorsal to the metapterygoid. This is a smooth bone with a convex dorsal border. A small gap appears between the ventral border of the dermohyomandibular and the dorsal border of the preoperculum. This suggests that the preoerculum was displaced ventrally during fossilization and would have been located directly ventral to the dermohyomandibular in life.

The large hollow between the preoperculum and the cleithrum also suggests anterior displacement of the preoperculum. In between the dermo-hyomandibular/preoperculum complex and the cleithrum would have been the operculum. However, there is no evidence of an operculum preserved on this specimen.

A bone that is interpreted to be a branchiostegal ray is located ventral to the antero-ventral margins of the preoperculum/dermohyomandibular complex. It is a singular ray and is heavily disarticulated. The anterior margin is flat with a slightly crushed antero-ventral border due to preservation bias. The dorsal margin is concave while the ventral margin is slightly convex. The rocky matrix overlies the posterior end of the bone so the true length of the bone is unknown, but the width of the ray is broad.

##### Pectoral girdle

A narrow, slightly crescent-shaped bone found posterior to the probable preoperculum/hyomandibular complex is most likely the cleithrum (Fig. 1). The cleithrum is a very large bone, which encircles the whole opercular skeleton (Fig. 1). Much of this bone is obscured by rocky matrix, making its morphology difficult to discern. The dorsal margin is posterior to the orbit. The bone widens to a large medial extension and a similar extension is found ventral to the opercular skeleton. Some parts of the bone that are exposed are located postero-medially and are ornamented with pits and ridges, the latter becoming more common closer to the posterior margins of the cleithrum.

The antero-dorsal arm of the cleithrum is found posterior to the caudal plate of the orbit. This arm is ornamented with small vertical ridges and pits. Two ridges are present along the outer margins throughout the entire bone. The ventral tip of this arm appears to be pointed judging by how the bone narrows towards the tip, but the rocky matrix between the sutures obscures this area. The cleithrum arm broadens towards the dorsal margin. The dorsal margin appears to be fragmented but seems convex forming two bifurcations. It is dorsal to the dermo-hyomandibular. These are broad with rounded margins.

##### Axial skeleton

There are seven autogenous neural spines preserved (Fig. 1). They are located posterior to the post-parietal process, dorsal to the cleithrum and anterior to the preserved imprint of notochord. These spines are long, thin bones with a large anterior flange, which tapers to a point distally, but no tips of the autogenous neural spines are preserved. The first four spines are overlain by the tips of the dorsal flank scales, confirming the decrease in size of the scales as they continue into a cephalo-caudal direction.

There are 21 neural spines excluding those in the caudal peduncle (Fig. 1). All are preserved as imprints in the rock, with a single exception. This spine is the first neural spine in the series and it is located posterior to the last autogenous neural spine. It is an elongate and thin bone with a broad anterior flange.

No haemal spines are preserved as bone (Fig. 1). Twelve imprints of haemal spines are observed but since there is a 1:1 distribution of haemal to neural spines there is presumably a minimum of at least 21 haemal spines in this specimen. Anterior haemal spines are obscured by the rocky matrix and ventral flank scales. No articulation between the neural and haemal spines is preserved along the open notochordal canal. Although the arcocentra of neither haemal nor neural spines are preserved, the impressions on the fossil hint at these spines having an expanded base that would have contacted the arcocentra of the adjacent neural and haemal spines, suggesting that there was a simple contact between these arcocentra.

##### Dorsal and anal fins

All remains of both the dorsal and anal fin are preserved as imprints only (Fig. 1). The preserved dorsal fin is comprised of seven fin rays, which are supported by 11 axonosts. The dorsal fin faces in an anterior direction and the fin rays simultaneously increase in size in an anterior direction. The fin is greatly elongate and has a falcate shape. Due to the very low number of fin rays and the position of the fin, disarticulation seems likely. A large V-shaped gap is located between the neural spines underneath the first three dorsal axonosts. This gap is most likely due to disarticulation.

The anal fin is located posterior to the post-coelomic bone. It is comprised of six fin rays. The fin rays increase greatly in size in a cephalocaudal direction. The anal fin has an elongate, falcate shape similar to the dorsal fin. There is no evidence of further fin rays being preserved, which would make this a very distinctive anal fin for a pycnodont fish. The number of axonosts is unknown as they are not preserved.

##### Caudal endoskeleton and fin

The caudal peduncle contains four neural spines preserved as bone, and four haemal spines preserved as imprints in the rock (Figs 1, 4A). All neural spines curve backwards and have a large fan-shaped base with crenulations on its ventral margins. The second most posterior spine has a concave dorsal margin. The most posterior spine has a large anterior lateral process protruding from the antero-dorsal margin. No tips of spines are preserved, but the imprints in the rock show that they extended in a posterior direction and that the tips were as long as the remainder that was preserved.

The cast of the caudal fin imprint reveals additional details about its morphology (Fig. 4B, C). There is a large parhypural located anteriorly to the hypochordal series. It is a club-shaped bone with a large ventral arcocentrum that curves backwards and appears to have overlapped the base of the first hypochordal. There are 10 hypochordals in total. Hypochordal 1 has a large expanded head with a narrow body that is inclined in an anterior direction. Hypochordals 2 and 3 are spoon shaped, with hypochordal 3 being the longer of the two. Hypochordals 4–7 have narrow heads with rounded margins and are fan-shaped bones which show evidence of hypertrophy due to the presence of longitudinal crests. The head of hypochordal 6 is overlain by hypochordal 7 but it still retains the fan shape of the two preceding hypochordals. Hypochordal 7 appears to be a compound bone as it shows a separation of the bone medially and at the anterior margin but not at the base. Hypochordal 8 is fan shaped but narrower than the preceding fan-shaped hypochordals, while hypochordal 9 is significantly broader. Hypochordal 10 appears to have a similar shape to hypochordal 8 with the exception of having a broader base. There are no longitudinal crests present on hypochordals 8–10.

There are only four epichordals observed. They are located ventral to the fin rays and anterior to hypochordal 10. The imprints reveal that they were elongate, thin bones that reach at least to the antero-medial border of the last hypochordal in length. Epichordal 1 is the smallest of the bones while epichordals 2–3 are far longer, being twice the length of the first epichordal. Epichordal 4 is only moderately longer than the first epichordal.

No fin rays are preserved as bone (Figs 1, 4A). Thirty to 31 fin rays are preserved as imprints in the rock. The general shape of the fin suggests that dorsal and ventral outer fin rays extend outwards *c*. 3 cm from the caudal peduncle. The median fin rays barely extend outwards from the hypochordal bones (*c*. 4 mm). The imprint of the caudal fin margin suggests that it has a convex median distal border.

##### Post-coelomic bone

Located posterior to the cloaca is the post-coelomic bone (Figs 1, 5). The bone itself is not preserved but the shape is clearly imprinted into the rock. It is a long, thin bone, which broadens to a club near its distal tip. The anterior portion of the club-like bone has convex dorsal and ventral margins while the medial margin is flat. The posterior margin of this bone is flat. It can be seen that the bone curves in an anterior direction.

##### Squamation

Posterior to the dermosupraoccipital is a large spine (twice as tall as preceding ossification) with a broad base, which narrows in a ventro-dorsal direction towards the distal tip. The ridge present on its anterior margin disappears as it reaches the distal tip of the scale. The tip of the spine curves anteriorly with the dorsal and ventral margins having a convex contour and a concave contour, respectively. The spine is broken off at tip, which is displaced ventrally to the concave margin of the spine (Fig. 2A, C). The tip of the spine has a rounded margin. This spine represents the first dorsal ridge scale. This scale originates from the posterior base of the dermosupraoccipital where it is incorporated into the skull roof (Figs 1, 2A–C).

The second dorsal ridge scale shows that the posterior portion has a large rectangular margin. The first dorsal ridge scale also has this rectangular extension but it is overlain by a displaced scale. The second dorsal ridge scale also appears to be narrowing to a distal tip anteriorly but this is not observed directly, instead being deduced from the concave notch present in the anterior portion of the scale before it is broken off (Fig. 2D).

Three dorsal ridge scales are preserved as bone and six are preserved as imprints in the rocky matrix, making a total of nine scales (Fig. 1). The preserved scales posterior to the first dorsal ridge scale are smooth with a flat dorsal margin and no spines. The scale has a medial ridge surrounded by a posterior rectangular extension and a narrowing anterior portion with a concave notch. There is a long, thin pointed antero-ventral process protruding from the dorsal ridge scale while it overlies the flank scales. The few scales that are preserved in bone increase in width in a cephalo-caudal direction.

Postero-ventral to the cleithrum are the ventral ridge scales (Fig. 1). There is a total of 10 scales preserved in bone. Like their dorsal ridge scale counterparts, an increase in scale width can be observed in a cephalo-caudal direction. The best-preserved scale is located posterior to the cloaca. It has a smooth surface and two spines not in contact with each other. Flank scales located anterior to the cloaca are slimmer in width, and two appear dorsal to the anterior and posterior ends of the ventral ridge scale. On the scales where the spines are preserved, there appears to be a maximum number of three. The spines are more visible in scales when observed in a cephalo-caudal direction.

The flank scales are thin, elongate scale bars that are ventral to the dorsal ridge scales and dorsal to the ventral ridge scales (Fig. 1). The dorsal flank scales have a medial ridge going down the length of the scale bar. The flank scales curve posteriorly. These flank scales decrease in length the closer they are to the dorsal fin. On the ventral side, flank scales are singular, thin and elongate ventral to the cleithrum. Posterior to the cleithrum, they become much broader with numerous ridges (usually two to three) on the surface. These reach the notochord and are not preserved in the medial portion of the fossil. No articulation is seen between the dorsal and ventral flank scales, so the number of complete scale rows remains unknown.

The cloaca is not roofed by a bifid scale. A fragment of the scale that forms the roof of the cloaca can be observed in an antero-ventral position. A slight concave notch is present ventrally on the preserved portion of the scale. No modified cloacal scales as originally described by Poyato-Ariza & Wenz (2002, p. 203) are present in this specimen. Instead, there are what appear to be bifurcating scales located anterior and posterior to the cloaca (Figs 1, 5). The anterior cloacal scale bifurcates in a vertical, slightly anterior direction. The left branch is ornamented with small ridges while the right branch is smooth. Bifurcation begins closer to the anterior portion of the scale that roofs the cloaca. The anterior margin is concave while the posterior margin is convex. The posterior cloacal scale begins to bifurcate vertically above the cloaca. The ventral base is broad and curves anteriorly revealing a convex margin. Both branches are preserved as an imprint in the rocky matrix. The left branch is significantly longer than the right and becomes narrower at its tip. Bifurcation of the posterior cloacal scale is also preserved as an imprint. The true length of the right branch is unknown as the tip is overlain by disarticulated flank scales. We have reconstructed it to be of the same height as the left branch (Fig. 5B). Both bifurcated cloacal scales are dorsal to the ventral ridge scales. No scales are preserved posterior to the post-coelomic bone.

## Phylogenetic analysis

A phylogenetic analysis using the data matrix of Poyato-Ariza & Wenz (2002) was performed to establish the relationship of *Scalacurvichthys* gen. nov. to other pycnodonts. All characters were treated as unordered and unweighted. The branching scales located anterior and posterior to the cloaca are interpreted here as modified cloacal scales, which are characters 102 and 103 in the original data matrix of Poyato-Ariza & Wenz (2002). The character coding for Pycnodontiformes and, in particular, *Scalacurvichthys naishi* gen. et sp. nov., and the modified data matrix are available in the Supplemental material.

A total of 32 most parsimonious trees (MPTs) were produced. The strict consensus tree (SCT) has a consistency index (CI) of 30, a retention index (RI) of 11 and a tree length of 752 steps. The majority rule consensus tree (MRCT), conversely, has a CI of 46, an RI of 54 and a tree length of 501 steps.

**Figure 6.**
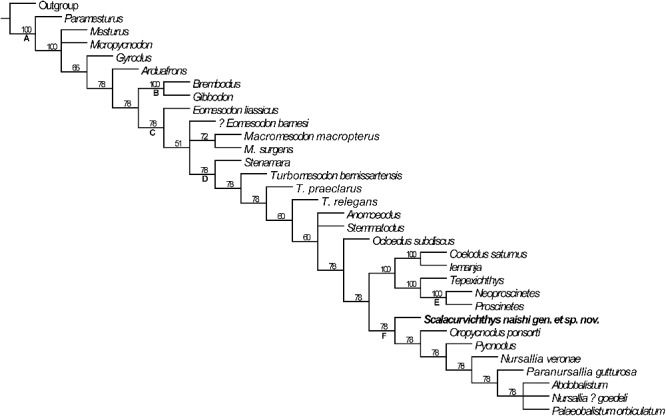
Majority rule consensus tree depicting the systematic position of *Scalacurvichthys naishi* gen. et sp. nov. holotype (SMNK-PAL. 8613) with all unordered characters based on modified database of Poyato-Ariza & Wenz (2002). Nodes are as follows: A, Pycnodontiformes; B, Brembodontidae; C, Pycnodontoidei; D, Pycnodontidae; E, Proscinitinae; F, Pycnodontinae.

The results of the phylogenetic analysis revealed consistently that *Scalacurvichthys naishi* is the sister taxon to a clade comprising *Oropycnodus* from the Paleocene (Montian) of Mont Aimé, Chalons-sur-Marne, France, and the remaining pycnodontins (Poyato-Ariza & Wenz 2002) (Fig. 6). The autapomorphic characters of *Scalacurvichthys naishi* are: (52:3) number of vertebrae: 24 or less; and (101:6) number of post-cloacal ventral keel scales: one.

## Discussion

The phylogenetic analysis reveals that *Scalacurvichthys naishi* gen. et sp. nov. is sister to (*Oropycnodus* + (*Pycnodus* + (*Nursallia veronae* + (*Paranursallia gutturosum* + (*Abdobalistum* + *Nursallia*? *goedeli* + *Palaeobalistum orbiculatum*))))). This arrangement unambiguously supports *Scalacurvichthys naishi* as a member of the subfamily Pycnodontinae.

According to Poyato-Ariza & Wenz (2002), the two main autapomorphies of Pycnodontinae are the presence of a posteriorly exposed endocranium with a postcephalic lacuna (17:1) and scutellum-like contour scales present only dorsally (70:1). In *Scalacurvichthys*, the posteriorly exposed endocranium is well discernible under UV lighting even though it is very incompletely preserved. The post-cephalic lacuna is either not preserved or not developed. However, even if *Scalacurvichthys naishi* lacked a postcephalic lacuna, the presence of a posteriorly exposed endocranium would still include *Scalacurvichthys naishi* into Pycnodontinae, as is the case for *Sylvienodus* (Poyato-Ariza 2013) and *Polazzodus* (Poyato-Ariza 2010). The contour scales are not scutellum shaped, particularly those on the dorsal region. Consequently, character 87 (scutellum-like contour scales) is marked as [0] (not differentiated). All the other possible pycnodontin characters of Poyato-Ariza & Wenz (2002) observed in *Scalacurvichthys* are homoplastic. These are characters 88 (7 to 9 dorsal ridge scales, [4]), 94 (10 to 14 ventral keel scales, [4]) and 97 (spines on ventral keel scales in the posterior region of the midline, [2]).

According to the results from the phylogenetic analyses, *Scalacurvichthys naishi* is supported as a new genus based on the following autapomorphic characters: (1) less than 24 (21) vertebrae; (2) a single post-cloacal ventral ridge scale present.

Additionally, its morphology is highly distinctive as *Scalacurvichthys* possess a large triangular dermocranium, large anteriorly curved first dorsal ridge scale which protrudes above the skull roof and large, anterior and posterior bifurcating cloacal scales. The skull shape of *Scalacurvichthys naishi*, for example, differs from that of *Pycnodus* and *Oropycnodus* in having a sharply sloping forehead, whereas it is drastically antero-posteriorly shortened in contrast to the elongated skull of *Polazzodus*. The first dorsal ridge scale in *Scalacurvichthys naishi* is anteriorly curved and protrudes above the skull roof, in contrast to the low-lying first dorsal ridge scale of the closely related pycnodonts *Sylvienodus* and *Pycnodus*, and it additionally is narrower than the same scale on *Polazzodus*. The scutellum-like dorsal ridge scales seen in *Pycnodus* are absent in the new taxon described here. There is only a single post-cloacal ventral ridge scale, in contrast to two as in other pycnodontins such as *Pycnodus*, *Oropycnodus*, *Polazzodus* and *Sylvienodus*.

**Figure 7.**
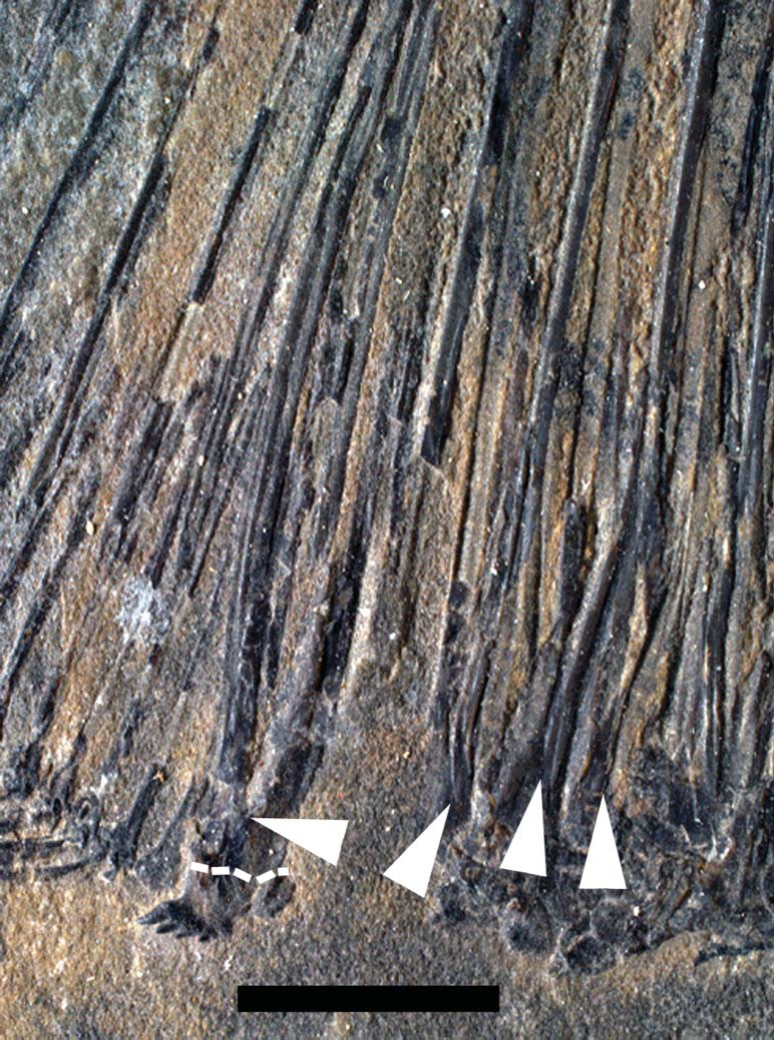
Cloacal region of *Stemmatodus rhombus* (Heckel, 1854) MNHN JRE 41 with the arrows pointing to the bifurcated scales anterior and posterior to the cloaca. Dashed line indicates boundary between ventral ridge scale and bifurcated posterior modified cloacal scale. Scale bar = 50 mm.

The bifurcating scales are especially notable in this genus, and the presence of bifurcated scales around the cloaca in two other pycnodontid genera suggests not only the discovery of a new character previously unknown in pycnodont fishes but that it could be more widespread throughout the family as a whole. Since this form of scale has not been described before in the literature in any other genera of Pycnodontidae, it is imperative to discover whether such a character has been undetected in other pycnodontids or if there is a true absence of these scales in these taxa. We identified bifurcated scales in at least two other pycnodontids. *Stemmatodus rhombus* Heckel, 1854 (MNHN JRE 41), for instance, has three bifurcating scales anterior to the cloaca (Fig. 7). The right arm of the most anterior scale is orientated in an antero-dorsal direction while the left arm is parallel to the right arm of the medial bifurcating scale. The left arm of the medial scale is broader than the right arm. In the most posterior of the bifurcating scale series, and thus the one directly anterior to the cloaca, bifurcation can be more difficult to see as the posterior margin of the base of the left arm has eroded away, but bifurcation can be observed in the ventro-medial portion of the scale. Both posterior and medial bifurcating scales branch off in an antero-dorsal direction. There is a single bifurcating scale located posterior to the cloaca and dorsal to the post-cloacal ventral ridge scale. Both arms branch vertically in a slight anterior direction. The right arm is twice the height of the left arm and they are both broad in width.

**Figure 8.**
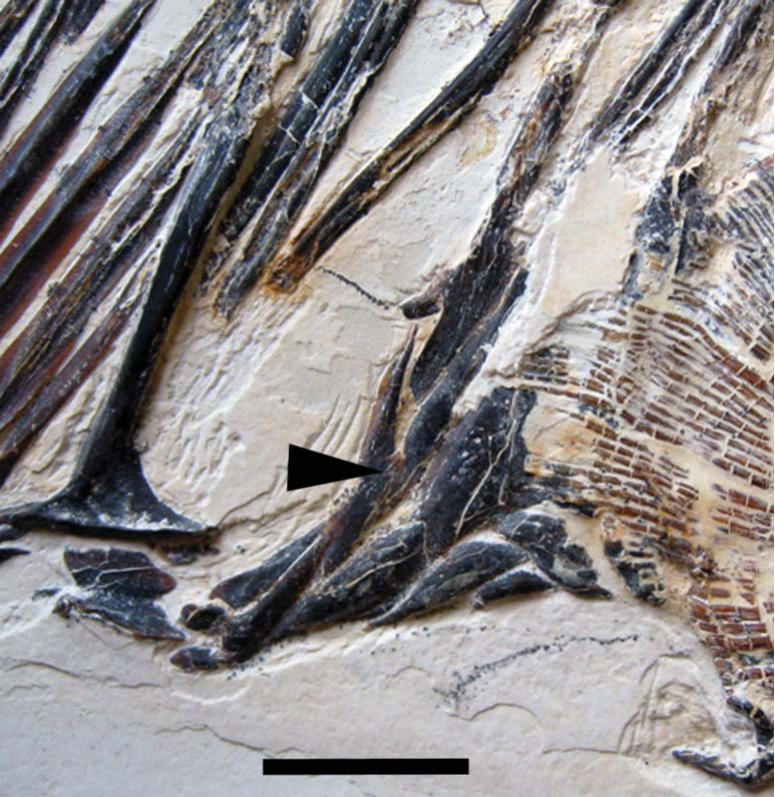
Cloacal region of *Proscinetes bernardi* (Thiollière, 1852) JME 250 with the arrow pointing to the bifurcating cloacal scale present on this specimen. Scale bar = 1 cm.

The second pycondontid taxon found to have bifurcating scales is *Proscinetes bernardi* Thiollière, 1852 (JME 250) (Fig. 8). Only one such scale is present in this specimen, which is caudal to the posterior modified cloacal scales, positioned among their dorsal margins. A posterior process with rounded margins protrudes from its base. The left branch is smaller than the right branch. The right branch is oriented in an anterior direction while the left branch is more vertical with the tip also oriented anteriorly. There is a concave posterior margin to the tip of the left branch while the tip is broken off on the right branch. It remains ambiguous whether there is a second bifurcating scale present on this specimen, because overlying pelvic fin rays obscure the anterior margin of the cloaca. Consequently, bifurcating scales seem not unique to *Scalacurvichthys* gen. nov., but their number and position in relation to the cloaca seemingly are unique for each pycnodont, and it is among the most distinctive autapomorphic characters found in the new taxon. Moreover, the dorsal and anal fins are extremely falcate in the new taxon, more so than those in *Sylvienodus*, and in contrast to *Pycnodus*, *Polazzodus* and *Oropycnodus*.

There are two major differences between the MRCT produced here and that of Poyato-Ariza & Wenz (2002). *Stenamara* and all members of *Turbomesodon* have also changed positions on the tree with *Stenamara* being sister to *Turbomesodon bernissartensis*. The clade supporting all species belonging to *Turbomesodon* (*Turbomesodon praeclarus +* (*T. bernissartensis* + *T. relegans*)) has broken down and now all members of *Turbomesodon* are sister to each other. It also shows that Pycnodontinae is a more inclusive subfamily than previously indicated (Poyato-Ariza & Wenz 2002, 2005; Ebert 2016), if all characters are treated as unordered. Nursallinae is found within Pycnodontinae instead of being a sister group to Pycnodontinae, a phylogenetic hypothesis that already was proposed by Taverne & Capasso (2012, fig. 13). Resolving these controversies is beyond the scope of this paper and will be addressed in detail elsewhere.

## Conclusions

Almost all Cenomanian fishes from Israel have been recorded either from late Cenomanian deposits of the Kefar Shaul Formation in the vicinity of Jerusalem (Blanckenhorn 1905; Shalem 1925, 1927; Raab & Chalifa 1987) or from the ‘Ein Yabrud quarries north of Jerusalem, which are early to middle Cenomanian in age (see above). The collection that originally was assembled by the Austrian zoologist and palaeontologist Georg Haas, who joined the Hebrew University in Jerusalem in 1932, comprises the oldest snakes known to date (e.g. Head 2015), pterosaur remains, turtles, lizards including mosasaurs, and hundreds of fish specimens (e.g. Polcyn *et al*. 1999). So far, only a fraction of the fossil fish assemblages have been studied in detail, including holosteans such as amiiforms (Chalifa & Tchernov 1982) and various teleosts such as dercetids, gonorhynchids, enchodontids, eurypholids, halecids, pharmacichthyids, aipichthyids and trachichthyoids comprising at least 13 species (see Khalloufi *et al*. 2010 for a complete list of species). The new pycnodont fish described here is the first representative of Pycnodontomorpha from the Cenomanian of Israel known to date. It is remarkable that no pycnodontiform fish from the ‘Ein Jabrud assemblages has been reported before. Deposition of the finely laminated limestones is considered to have been in a structured, near-coastal area characterized by reefs with interspersed lagoons, and pycnodontiform fishes mainly occupied such habitats (e.g. Kriwet 2001a, b). Consequently, we assume that *Scalacurvichthys naishi* gen. et sp. nov. was an endemic fish inhabiting the eastern margin of the Tethys that represented a NW-dipping carbonate platform delimiting the eastern margin of the Tethys (e.g. Powell 1989; Philip *et al*. 2000). A detailed study of the ‘Ein Yabrud fishes will surely contribute to our knowledge of pycnodontiform fishes of the Jordanian shelf.

Even though the exact geographical and stratigraphical provenance of the new taxon remains unknown, the lithology and colour of the limestone slab containing the specimen and its preservation support our assumption that the new taxon comes from one of the so-called ‘Ein Jabrud quarries close to the village of Beit El near Ramallah, north of Jerusalem, and thus is of early to middle Cenomanian age.

The new genus is quite peculiar and displays morphological features (e.g. bifurcating cloacal scales) that have not been recognized before, but seemingly also are present in other, well-known pycnodonts. *Scalacurvichthys naishi* is well supported as a member of the sub-family Pycnodontinae within the family Pycnodontidae, according to the results presented here. Nevertheless, the definition of Pycnodontinae is in need of further refinement due to *Sylvienodus* Poyato-Ariza, 2013 and *Polazzodus* Poyato-Ariza, 2010 not having the full suite of characters that diagnose the subfamily Pycnodontinae, and pending the discovery of further specimens of the new genus presented here. Further refinement may be acquired by revising many of these specimens, and the creation of a new character database for Pycnodontidae.

## Supplementary Material

Appendix_1_Cawley.pdf

Cawley_Kriwet_2017.nex

Character_matrix.pdf
