# Supplementary material for: A new pycnodont fish, Scalacurvichthys naishi gen. et sp. nov., from the Late Cretaceous of Israel
Source: J Syst Palaeontol. 2017 Jun 14;16(8):659–73. doi: 10.1080/14772019.2017.1330772 (PMC5849399; doi:10.1080/14772019.2017.1330772)
Supplement: Appendix_1_Cawley.pdf [file TJSP_A_1330772_SM2548.pdf]

Appendix 1

This is the list of characters taken from the matrix of Poyato-Ariza & Wenz (2002). Character states underlined are those that apply to *Scalacurvichtys naishii* nov gen et sp. Characters with no states underlined are unknown in the specimen of *Scalacurvichthys* being studied and are marked as “?” in the data matrix.

1. Body shape (as measured by the ratio maximum body height/standard length)

Fusiform, less than 40% (0); <u>intermediate, 40-70% (1)</u>; discoid, 70-100% (2); deep, more than 100% (3).

2. Relative position of dorsal apex

Apex absent (0); <u>before the point of insertion of the dorsal fin (1);</u> in the point of insertion of the dorsal fin (2).

3. Morphology of dorsal prominence

<u>Dorsal prominence absent (0);</u> pointed, posterior border inclined (1); obtuse angle, posterior border (sub)horizontal (2); curved, anteriorly oriented (3); curved, dorsally oriented (4).

4. Relative position of ventral apex

Apex absent (0); <u>before the point of insertion of the anal fin (1);</u> in the point of insertion of the anal fin (2).

5. Mouth gape

Horizontal or subhorizontal (0); <u>inclined (1);</u> subvertical, opening downward (2).

6. Prognathism

<u>Absent (0);</u> present (1).

7. Caudal pedicle

Differentiated (0); <u>not differentiated (1).</u>

8. Antorbital and ethmoidal regions

Normal (0); <u>hypertrophied (1).</u>

9. Morphology of parietal bones

Rectangular, long (0); curved, long (1); curved, short (2); curved, very broad (3).

10. Preparietal bones

Absent (0); present (1).

11. Parietal spine

Absent (0); present, simple (1); present, compound (2).

12. Dermocranial fenestra

Absent (0); present (1).

13. Post-parietal

Single (0); divided (1); absent (2).

14. Post-parietal process

Absent (0); present (1).

15. Dermal supraoccipital

Absent (0); single (1); divided into two or more unpaired plates (2).

16. Supraoccipital spine

Absent (0); present, simple (1); present, compound (2).

17. Extrascapulars hypertrophied

No (0); yes (1).

18. Extrascapular(s) fused to (post) parietal

No (0); yes (1).

19. Endocranium posteriorly exposed

No (0); yes (1).

20. Anterior portion of infraorbital sensory canal

Closely surrounding the orbit (0); descending towards the ethmoid region (1).

21. Infraorbitals

Row of plates around the ventral and posterior border of the orbit (0); mosaic of small plates partially covering the cheek (1); reduced to tubular ossifications around the infraorbital sensory canal (2); anterior infraorbital enlarged (3).

22. Infraorbital ornamentation

Present in all infraorbitals (0); present only in the posteriormost one (1); absent in all infraorbitals (2).

23. Suborbitals

One or several rows (0); mosaic of small plates (1); absent as independent ossifications (2).

24. Preopercular and hyomandibular

Preopercular single, smaller than opercular, hyomandibular deep, unornamented (0); preopercular single, hypertrophied, hyomandibular deep, unornamented (1); one large preopercular plus a small ornamented plate over the head of the hyomandibular (2); one large preopercular in close contact with a small ornamented portion of the hyomandibular, at the same superficial level (3); preopercular of similar size to expanded superficial ornamented portion of hyomandibular (4).

25. Opercular process of hyomandibular

Present, well developed (0); present, reduced (1); absent (2).

26. Condyle in articular head of hyomandibular

Absent (0); present (1).

27. Suboperculum and interoperculum

Present (0); absent (1).

28. Opercular bone

Well developed (0); reduced (1); extremely reduced (2).

29. Ossifications in gular region

Large gular plate (0); small, numerous tesserae (1); no ossifications (2).

30. Branchiostegal rays

More than two (0); two, relatively large, in contact (1); two, thin, separated (2).

31. Premaxillary process

Profound (0); anteriorly placed, long, superficial (1).

32. Morphology of premaxillary and dentary teeth

Small, triangular to conic (0); robust, columnar to hookshaped (1); robust, barely incisiform (2); very flattened, fully incisiform (3).

33. Crown of premaxillary and dentary teeth

Simple (0); bifurcated (1).

34. Number of premaxillary teeth

More than three (0); three (1); two (2).

35. Maxilla

Teeth-bearing, ornamented, elongated (0); edentulous, ornamented, ovoid (1); edentulous, ornamented, elongated (2); edentulous, unornamented, reniform (3); edentulous, unornamented, straight oral border (4); edentulous, unornamented, elongated oval (5).

36. Morphology of vomerine teeth

Villiform to conic (0); circular to subcircular contour (1); oval contour (2); reniform contour (3); triangular contour (4).

37. Arrangement of vomerine teeth in regular rows

Absent (0); present (1); absent anteriorly, present posteriorly (2).

38. Number of vomerine tooth rows

Not arranged in rows (0); three (1); five (2).

39. Number of teeth in principal vomerine tooth row

Teeth not arranged in rows (0); seven or less (1); eight or nine (2); 10 or more (3).

40. Alternation of teeth on main vomerine tooth row

Absent (0); present (1).

41. Dentary

Well developed, relatively broad (0); small, posteriorly elongated and simple (1); small, posteriorly bifid (2).

42. Number of dentary teeth

More than five (0); five (1); four (2); three (3); two (4).

43. Morphology of prearticular teeth

Villiform to conic (0); circular contour (1); oval contour (2); sigmoid to drop-shaped contour (3); extremely elongated in contour (4).

44. Arrangement of prearticular teeth in regular rows

Absent (0); present (1); absent anteriorly, present posteriorly (2).

45. Number of prearticular tooth rows

Not arranged in rows (0); two (1); three (2); four (3); five or six (4).

46. Number of teeth on main prearticular tooth row

Teeth not arranged in rows (0); seven or less (1); eight or nine (2); 10 or more (3).

47. Coronoid process

Low, curved (0); high, straight dorsal border (1); high, club-shaped (2); low, straight dorsal border (3).

48. Central papilla in vomerine and prearticular teeth

Absent (0); present (1).

49. Crenulations in vomerine and prearticular teeth

Absent (0); occasionally present, weak (1); present in most teeth, strong (2).

50. Ridge on vomerine and prearticular teeth

Absent (0); present (1).

51. Groove on vomerine and prearticular teeth

Absent (0); present (1).

52. Number of vertebrae

35 or more (0); 30-34 (1); 25-29 (2); 24 or less (3).

53. Neural and haemal corresponding arcocentra

Not surrounding notochord (0); surrounding notochord partially (1); surrounding notochord completely (2).

54. Neural and haemal adjacent arcocentra

Separated from each other (0); simple contact (1); complex contact (2); hyper-complex contact (3); expanded and imbricate (4).

55. Sagittal flanges on neural and haemal spines

Absent (0); anterior, small and short (1); anterior, large and long (2); anterior and posterior (3); anterior and posterior with strengthened margins (4).

56. Number of autogenous anterior neural spines

Outgroup (?); most of them, including caudal ones (1); 10 or more (2); seven to 10 (3); six or less (4).

57. Relative length of last neural spine not supporting precurrent caudal fin rays

Outgroup (?); reduced (1); less than half as long as preceding spines (2); vestigial (3).

58. Number of epichordal elements of caudal endoskeleton

Nine or more (0); six to eight (1); four or five (2); three (3).

59. Relative development of hypochordal elements of caudal endoskeleton

Only slightly enlarged (0); enlarged, plate-like (1); hypertrophied (2).

60. Number of hypochordal elements of caudal endoskeleton

14 or more (0); 12-13 (1); 9-11 (2); six to eight (3).

61. Diastema

Absent (0); present (1).

62. Cleithrum

Two limbs in angle, anteroventral limb subhorizontal (0); curved, anteroventral limb subhorizontal, slightly expanded (1); curved, anteroventral limb subvertical, expanded (2); cleithrum with three limbs (3); cleithrum with four limbs (4).

63. Spines on cleithrum

None (0); 1, hypertrophied (1); about 10 (2); about 50 (3).

64. Position of pelvic fins (ratio prepelvic distance/standard length)

45-55% (0); more than 55% (1); less than 45% (2).

65. Position of dorsal fin (predorsal length/standard length)

60%-69% (0); 40%-49% (1); 50%-59% (2); 70%-79% (3).

66. Number of dorsal axonosts

Less than 20 (0); 20-29 (1); 30-39 (2); 40-49 (3); 50-59 (4); 60 or more (5).

67. Dorsal axonost not supporting lepidotrichium (free axonost)

Absent (0); present (1).

68. Morphology of the dorsal and anal fins

Strip-like (0); falcate to acuminate (1); sigmoid outline (2); rounded in the centre (3); rounded anteriorly (4); square (5).

69. Position of anal fin (preanal length/standard length)

70%-79% (0); 50%-59% (1); 60%-69% (2); 80%-89% (3).

70. Number of anal axonosts

10-19 (0); 20-29 (1); 30-39 (2); 40-49 (3); 50 or more (4); nine or less (5).

71. Urodermals

Not differentiated (0); a series of three or more (1); two (2); one (3); absent (4).

72. Number of caudal principal fin rays

20-25 (0); nine or less (1); 10-19 (2); 26-35 (3); 36 or more (4).

73. Morphology of caudal fin

Outgroup (?); stalked (1); distal border convex (2); distal border concave (3); distal border straight (4); double emarginated (5); vertical (6).

74. Fringing fulcra

Present, numerous (0); present, scarce (1); absent (2).

75. Ossification of scales

Complete in all scales (0); complete in abdominal scales, incomplete in caudal scales (1); complete in ventral scales, incomplete in dorsal scales (2); incomplete in all scales (3); scales absent (4).

76. Distribution of scales

Whole body (0); whole body except caudal pedicle (1); abdominal region plus part of the caudal region (2); only abdominal region (3); body naked (4).

77. Arrangement of scales

Rows in the same direction (0); rows in different directions (1); not forming rows (2); scales absent (3).

78. Suture between scales of the same row

Not jagged (0); jagged (1).

79. Scale rows

Simple (0); double (1).

80. Scale rows between the bases of the lepidotrichia of the dorsal and anal fins

Absent (0); present (1).

81. Ornamentation

Outgroup (?); ridges (1); reticulation (2); tubercles (3); small spines (4).

82. Large spines on scales

None (0); one (1); several (2).

83. Nuchal plates

Absent (0); present (1).

84. Dorsal spine

Absent (0); present (1).

85. Contour scales

Not differentiated (0); differentiated (1); absent (2).

86. First dorsal ridge scale

Not differentiated (0); about same size than subsequent ridge scales (1); larger than subsequent ridge scales (2); absent (3).

87. Scutellum-like contour scales

Not differentiated (0); present, dorsal only (1); present, ventral only (2); present, dorsal and ventral (3); contour scales secondarily lost (4).

88. Number of differentiated dorsal ridge scales

Dorsal contour scales not differentiated (0); 18 or more (1); 15 to 17 (2); 10 to 14 (3); seven to nine (4); one or two (5); dorsal contour scales absent (6).

89. Arrangement of dorsal ridge scales

Dorsal contour scales in close contact with each other (0); point contact (1); separated from each other (2); dorsal contour scales absent (3).

90. Number of spines on dorsal ridge scales

No spines on dorsal contour scales (0); one or two (1); three or four (2); five or more (3); midline serrated (4); dorsal contour scales absent (5).

91. Distribution of spines on dorsal ridge scales

No spines on dorsal contour scales (0); all along the midline, or centered if only one spine present (1); posterior region (at most two thirds) of the midline (2); anterior region (at most two thirds) of the midline (3); dorsal contour scales absent (4).

92. Contact of spines on each dorsal ridge scale

No spines on dorsal contour scales (0); separated from each other (1); in contact with each other (2); dorsal contour scales absent (3).

93. Relative size of anterior and posterior spines on each dorsal ridge scale

No spines on dorsal contour scales (0); similar size (1); spines of increasing size in cephalocaudal sense (2); dorsal contour scales absent (3).

94. Number of ventral keel scales

Not differentiated (0); 22 or more (1); 18 to 21 (2); 15 to 17 (3); 10 to 14 (4); two or three (5); ventral keel scales absent (6).

95. Arrangement of ventral keel scales

Close contact with each other (0); point contact (1); ventral keel scales absent (2).

96. Number of spines on each ventral keel scale

No spines on ventral keel scales (0); one to three (1); four to six (2); seven or more (3); ventral keel scales absent (4).

97. Distribution of spines on ventral keel scales

No spines on ventral keel scales (0); all along the midline, or centered if only one spine present (1); posterior region (at most two thirds) of the midline (2); ventral keel scales absent (3).

98. Contact of spines on each ventral keel scale

No spines on ventral keel scales (0); separated from each other (1); in contact with each other (2); ventral keel scales absent (3).

99. Relative size of anterior and posterior spines on each ventral keel scale

No spines on ventral keel scales (0); all spines of similar size (1); spines of increasing size in cephalocaudal sense (2); ventral keel scales absent (3).

100. Several scales attached to the contour scales

No (0); yes (1); contour scales absent (2).

101. Number of post-cloacal ventral keel scales

Cloacal and contour scales not differentiated (0); 10 or more (1); seven or eight (2); five or six (3); three or four (4); two (5); one (6); none (7).

102. Number of anterior cloacal modified scales

Cloacal scales not modified (0); mosaic of little scales (1); two (2); one (3); cloacal scales absent (4).

103. Number of posterior cloacal modified scales

Cloacal scales not modified (0); mosaic of little scales (1); three (2); two (3); one (4); no scales, posterior part of anal notch supported by a rib (5); cloacal scales absent (6).

104. Bifid scale in cloaca

Absent (0); present (1); present plus several comma shaped scales (2).

105. Post-cloacal notch

Absent (0); present (1)
