# Supplementary material for: A new pycnodont fish, Scalacurvichthys naishi gen. et sp. nov., from the Late Cretaceous of Israel
Source: J Syst Palaeontol. 2017 Jun 14;16(8):659–73. doi: 10.1080/14772019.2017.1330772 (PMC5849399; doi:10.1080/14772019.2017.1330772)
Supplement: Character_matrix.pdf [file TJSP_A_1330772_SM2544.pdf]

**# characters**	**1**	**2**	**3**	**4**	**5**	**6**	**7**	**8**	**9**	**10**	**11**	**12**	**13**	**14**	**15**	**16**	**17**	**18**	**19**	**20**	**21**	**22**	**23**	**24**	**25**	**26**	**27**	**28**	**29**	**30**	**31**	**32**	**33**	**34**	**35**
Outgroup	0	0	0	0	0	0	0	0	0	0	0	0	0	0	0	0	0	0	0	0	0	0	0	0	0	0	0	0	0	0	0	0	0	0	0
Abdobalistum	2	1	0	?	0	0	1	1	?	?	0	0	?	1	1	0	?	?	0	1	3	?	?	?	?	?	?	1	2	?	1	2	0	?	?
Anomoeodus	?	?	?	?	?	?	?	?	?	?	?	?	?	?	?	?	?	?	?	?	?	?	?	?	?	?	?	?	?	?	?	3	0	?	?
Arduafrons	2	1	1	1	0	1	0	1	?	0	0	0	?	0	1	0	0	0	0	1	1	0	1	1	?	?	1	1	1	?	1	1	0	2	1
Brembodus	2	1	2	1	0	0	0	1	1	0	0	0	0	0	1	0	0	?	0	1	1	0	1	1	?	0	1	1	1	1	1	2	0	1	?
Coelodus saturnus	1	2	0	?	0	0	0	1	?	0	?	?	?	1	?	?	?	?	0	?	?	?	2	?	?	?	?	?	?	?	1	3	0	?	?
?Eomesodon barnesi	2	1	4	2	0	1	?	1	?	0	0	0	0	0	1	0	?	?	0	?	?	?	?	1	?	?	?	1	2	?	?	?	?	?	?
Eomesodon liassicus	2	1	3	1	0	?	0	1	1	0	0	0	0	0	1	0	0	1	0	?	0	0	?	1	?	?	1	1	?	2	?	2	0	?	?
Gibbodon	2	1	1	1	0	0	0	1	0	0	0	0	0	0	1	0	0	1	0	0	0	0	2	1	?	?	1	1	0	0	0	2	1	1	?
Gyrodus	2	0	0	1	0	0	0	1	1	0	0	0	0	0	1	0	0	0	0	0	1	0	1	1	1	0	1	1	1	2	1	1	0	2	1
Iemanja	1	?	0	?	0	1	1	1	2	0	0	0	0	1	1	0	?	?	0	?	?	?	?	3	2	1	1	1	2	?	1	?	?	?	5
Macromesodon macropterus	2	1	2	0	1	1	1	1	2	0	0	0	0	0	1	0	0	0	0	?	[12]	?	?	1	?	?	1	1	2	2	1	2	0	2	?
M. surgens	3	1	1	1	1	1	1	1	2	0	0	0	0	0	1	0	0	1	0	1	2	?	?	1	?	0	1	1	2	2	1	2	0	2	?
Mesturus	1	0	0	0	0	0	0	1	2	0	0	0	1	0	2	0	0	0	0	0	1	0	1	2	?	0	1	1	1	0	1	1	0	[12]	2
Micropycnodon	?	?	?	?	?	0	?	1	2	?	0	0	0	0	2	0	0	0	0	1	1	0	?	1	1	0	1	1	1	?	?	?	?	?	?
Neoproscinetes	2	1	0	1	1	0	0	1	2	0	0	0	0	1	1	0	0	?	0	1	2	1	2	3	2	1	1	1	2	2	1	3	0	2	3
Nursallia ?goedeli	?	?	?	?	?	?	?	?	?	?	?	?	?	?	?	?	?	?	?	?	?	?	?	?	?	?	?	?	?	?	?	?	?	?	?
Nursallia veronae	2	2	0	0	0	0	0	1	3	?	0	0	0	1	1	0	?	?	0	?	?	?	?	?	?	?	?	1	?	?	1	2	0	2	?
Ocloedus subdiscus	2	2	0	2	1	0	1	1	2	0	0	1	0	1	1	0	0	1	0	1	2	1	2	4	?	0	1	1	2	2	1	3	0	2	?
Oropycnodus ponsorti	2	2	0	1	0	0	1	1	2	0	0	1	0	1	1	0	0	1	1	1	2	?	2	4	?	?	1	2	2	?	1	3	0	2	?
Palaeobalistum orbiculatum	1	0	0	0	0	0	0	1	?	?	0	?	0	?	?	0	0	1	0	?	1	0	?	?	?	?	?	1	?	?	?	?	?	?	?
Paramesturus	?	?	?	?	0	0	?	1	2	0	0	0	0	0	2	0	0	0	0	0	0	?	?	?	?	0	?	?	?	?	1	1	0	?	?
Paranursallia gutturosa	2	2	0	0	0	0	0	1	3	1	0	0	0	?	1	0	0	0	0	0	3	0	2	3	?	0	1	1	2	?	1	2	0	2	1
Proscinetes	2	2	0	2	1	0	1	1	2	0	0	0	0	1	1	0	0	1	0	1	2	1	2	3	2	0	1	1	2	1	1	3	0	2	?
Pycnodus	1	1	0	0	0	0	0	1	2	0	0	1	0	1	1	0	0	0	1	1	3	0	2	4	2	0	1	1	2	2	1	3	0	2	3
Stemmatodus	1	1	0	0	1	0	1	1	2	0	0	0	0	1	1	0	0	?	0	1	2	1	2	4	2	0	1	1	2	2	1	3	0	2	4
Stenamara	3	1	4	1	1	0	1	1	2	0	0	0	0	1	1	0	0	?	0	1	2	?	?	?	?	0	?	1	?	?	?	?	?	?	?
Tepexichthys	2	2	0	2	1	0	1	1	2	0	0	1	0	1	1	0	0	1	0	1	2	2	2	?	?	?	1	1	2	?	1	3	0	1	3
Turbomesodon bernissartensis	2	1	0	1	1	0	1	1	2	0	0	0	0	1	1	0	?	?	0	?	?	?	?	?	?	?	?	1	?	?	1	3	0	2	?

T. praeclarus	2 1 0 1 1 0 1 1 2 0 0 0 0 1 1 0 0 1 0 1 2 1 2 3 2 ? 1 1 2 ? 1 3 0 2 ?
T. relegans	2 2 0 0 1 0 1 1 2 0 0 0 0 1 1 0 0 1 0 1 2 1 2 3 2 0 1 1 2 ? 1 3 0 2 3
Scalacurvichthys naishii nov. gen., n. sp.	1 1 0 1 1 0 1 1 2 0 0 0 0 1 1 0 0 0 1 ? ? ? 2 2 ? ? 1 ? 2 ? 1 3 0 2 ?

36	37	38	39	40	41	42	43	44	45	# characters	46	47	48	49	50	51	52	53	54	55	56	57	58	59	60	61	62	63	64	65	66	67	68	69	70
0	0	0	0	0	0	0	0	0	0	Outgroup	0	0	0	0	0	0	0	0	0	0	?	0	0	0	0	0	0	0	0	0	0	0	0	0	0
?	1	?	?	?	?	?	2	1	2	*Abdobalistum*	?	?	0	1	0	1	0	1	2	2	4	2	?	2	2	0	2	0	?	0	5	0	0	1	4
?	?	?	?	?	?	?	3	2	4	*Anomoeodus*	3	?	0	1	0	1	?	0	1	2	?	?	?	?	?	0	?	?	?	?	?	?	?	?	?
2	1	?	3	0	?	2	2	?	?	*Arduafrons*	3	0	0	0	0	0	0	0	0	0	?	?	?	?	?	?	1	0	0	3	3	0	0	0	2
2	1	2	2	0	1	2	2	1	4	*Brembodus*	2	1	0	0	0	0	2	2	1	1	1	1	2	0	3	0	2	0	1	1	2	1	[14]	0	1
2	1	2	3	0	?	?	4	1	2	*Coelodus saturnus*	3	?	0	0	0	1	1	1	1	2	3	1	?	2	2	1	2	0	?	?	5	0	1	?	3
?	?	?	?	?	?	?	2	1	4	*?Eomesodon barnesi*	2	1	0	1	0	0	?	0	0	1	?	?	?	0	?	0	2	0	2	3	2	0	3	2	1
?	?	?	?	?	?	4	2	?	?	*Eomesodon liassicus*	?	1	0	1	0	0	2	0	0	?	?	1	3	0	0	0	?	0	?	?	?	?	?	?	0
2	1	1	2	0	1	1	?	?	?	*Gibbodon*	?	?	0	1	0	0	?	?	?	?	?	?	?	?	?	?	2	0	1	0	0	0	0	0	5
1	1	2	3	0	2	2	2	1	3	*Gyrodus*	2	1	1	2	0	1	1	0	1	4	2	1	1	1	2	0	2	0	2	0	2	0	1	0	1
3	2	2	?	0	?	?	1	0	?	*Iemanja*	?	1	0	0	0	0	2	1	4	3	3	1	2	1	3	0	2	0	?	0	3	?	?	2	3
1	1	?	3	0	?	?	2	1	2	*Macromesodon macropterus*	2	?	0	1	0	0	0	0	0	1	?	1	3	0	1	0	2	0	0	0	2	0	4	0	0
2	1	2	?	0	1	2	2	1	?	*M. surgens*	?	1	0	1	0	0	0	0	0	1	?	1	3	0	2	0	2	0	0	0	2	[01]	4	0	1
1	1	2	3	0	2	[12]	1	1	4	*Mesturus*	3	1	0	2	1	1	1	0	?	?	?	?	?	?	?	?	1	0	?	0	2	0	0	2	1
2	1	2	1	0	?	?	2	1	3	*Micropycnodon*	1	2	0	2	0	1	?	?	?	?	?	?	?	?	?	?	?	0	?	?	?	?	?	?	?
2	1	1	2	1	?	3	2	1	2	*Neoproscinetes*	1	2	0	0	0	0	1	1	2	2	3	2	2	1	3	0	2	0	0	2	3	?	0	2	2
?	?	?	?	?	?	?	?	?	?	*Nursallia ?goedeli*	?	?	?	?	?	?	?	2	3	2	?	2	2	2	3	?	?	?	?	?	?	?	1	?	4
1	1	1	2	0	1	4	?	1	?	*Nursallia veronae*	2	?	0	0	0	0	2	2	3	?	?	3	2	2	3	0	2	0	0	2	5	0	1	2	4
2	1	2	3	0	1	4	2	1	2	*Ocloedus subdiscus*	2	1	0	1	0	1	2	0	1	2	3	1	2	1	1	0	2	0	1	2	2	0	1	2	2
2	1	?	1	0	1	4	2	1	2	*Oropycnodus ponsorti*	1	1	0	1	0	0	2	2	2	2	4	1	1	2	2	0	2	0	0	2	5	0	4	2	4
1	1	?	2	0	?	?	2	1	2	*Palaeobalistum orbiculatum*	2	?	0	0	0	0	?	?	?	?	?	?	?	1	2	0	?	0	0	0	?	?	?	1	?
?	1	?	3	?	?	2	?	?	?	*Paramesturus*	?	?	?	?	?	?	?	?	?	?	?	?	?	?	?	?	?	0	?	?	?	?	?	?	?
1	1	2	1	0	1	4	2	1	2	*Paranursallia gutturosa*	2	1	0	0	0	0	2	2	3	2	4	3	2	2	3	0	2	0	0	2	5	0	1	2	4
2	1	1	2	1	1	4	2	1	2	*Proscinetes*	2	1	0	0	0	0	1	0	1	2	3	1	2	1	2	0	2	0	1	2	[34]	1	1	2	3
1	1	2	2	0	1	4	2	1	2	*Pycnodus*	3	1	0	0	0	0	2	2	2	2	4	3	1	2	2	0	2	0	0	1	4	0	0	1	3
1	1	2	2	0	1	4	2	1	2	*Stemmatodus*	3	1	0	1	0	0	2	0	1	2	4	1	2	0	2	0	2	0	0	0	2	1	0	2	2
?	?	?	?	?	?	?	2	1	1	*Stenamara*	2	1	0	?	0	0	2	0	1	2	3	1	3	0	3	0	2	0	2	0	2	1	?	2	1
1	1	2	3	0	1	3	2	1	2	*Tepexichthys*	2	2	0	2	0	0	1	0	1	2	3	1	1	1	2	0	2	0	0	2	4	0	1	1	2
2	1	?	3	?	?	?	2	1	2	*Turbomesodon bernissartensis*	2	?	0	0	0	0	2	0	1	2	?	1	3	0	2	0	?	0	1	1	2	?	3	0	1

?	1	?	3	0	1	4	2	1	2	*T. praeclarus*	3	2	0	1	0	0	2	0	1	2	3	1	3	0	2	0	2	0	0	2	2	1	3	0	1
?	1	?	2	0	1	4	2	1	2	*T. relegans*	2	1	0	0	0	0	2	0	1	2	3	1	3	0	2	0	2	0	0	2	[23]	1	2	0	2
2	1	?	1	0	1	4	2	1	2	*Scalacurvichthys naishii* nov. gen., n. sp.	1	2	0	1	0	0	3	0	1	2	3	1	2	2	2	0	?	0	?	0	0	0	?	0	?

71	72	73	74	75	76	77	78	79	80	81	82	83	84	85	# characters	86	87	88	89	90	91	92	93	94	95	96	97	98	99	100
0	0	?	0	0	0	0	0	0	0	?	0	0	0	0	Outgroup	0	0	0	0	0	0	0	0	0	0	0	0	0	0	0
4	4	2	2	3	2	1	0	?	0	?	0	0	0	1	*Abdobalistum*	2	2	2	0	1	1	1	0	3	0	0	0	0	0	1
?	?	?	?	2	3	0	0	0	0	2	0	?	?	1	*Anomoeodus*	?	0	?	?	?	?	?	?	?	0	1	1	2	2	0
0	3	4	2	0	0	0	0	1	1	3	0	1	0	1	*Arduafrons*	1	0	1	0	3	1	1	1	1	0	3	1	1	2	0
1	2	2	0	0	0	0	0	0	1	3	0	1	1	1	*Brembodus*	1	0	3	0	3	1	1	1	2	0	?	?	?	?	0
?	0	5	2	2	3	0	0	0	0	?	3	?	0	1	*Coelodus saturnus*	?	?	3	2	0	0	0	0	?	?	?	?	?	?	0
?	?	?	2	0	3	0	0	0	0	3	0	1	0	1	*?Eomesodon barnesi*	1	0	2	0	?	?	?	?	?	0	?	?	?	?	?
?	?	?	2	0	3	0	0	0	0	3	0	?	0	1	*Eomesodon liassicus*	?	0	?	0	3	1	1	1	?	0	0	0	?	?	0
0	2	?	0	0	0	0	0	0	1	3	0	1	0	1	*Gibbodon*	1	0	3	0	2	1	1	1	2	0	3	1	1	?	0
3	0	1	2	0	0	0	0	0	1	3	0	0	0	1	*Gyrodus*	1	0	2	0	3	1	1	1	1	0	2	1	1	1	0
3	0	?	2	2	3	0	0	0	0	1	0	?	0	1	*Iemanja*	?	0	?	0	?	?	?	?	?	?	?	?	?	?	0
1	2	4	1	0	3	0	0	0	0	[34]	0	1	0	1	*Macromesodon macropterus*	1	0	1	0	3	1	1	1	1	0	2	1	1	2	0
1	2	2	2	0	3	0	0	0	0	3	0	1	0	1	*M. surgens*	1	0	1	0	2	1	1	1	2	0	3	1	1	2	0
0	3	2	1	0	0	1	1	1	1	3	0	0	0	1	*Mesturus*	2	0	1	0	2	1	1	1	1	0	3	1	1	1	0
?	?	?	?	0	?	0	0	0	?	3	0	?	?	1	*Micropycnodon*	2	0	?	?	?	?	1	?	?	0	2	1	1	1	?
3	0	3	2	2	3	0	0	0	0	1	0	?	0	1	*Neoproscinetes*	?	0	3	2	1	3	1	2	2	1	2	2	1	?	0
2	3	6	2	0	1	1	0	?	0	3	0	?	?	?	*Nursallia ?goedeli*	?	?	?	?	?	?	?	?	?	?	?	?	?	?	?
4	3	6	2	1	2	0	0	0	0	[13]	0	?	0	1	*Nursallia veronae*	2	3	3	1	0	0	0	?	4	1	?	?	?	?	0
2	2	5	2	2	3	0	0	0	0	3	0	0	0	1	*Ocloedus subdiscus*	1	0	3	1	2	2	2	2	3	0	3	2	2	2	0
3	0	5	2	3	3	0	0	0	0	[123]	0	0	0	1	*Oropycnodus ponsorti*	2	1	4	1	0	0	0	0	4	1	2	2	2	2	0
?	3	?	2	0	0	1	0	1	0	3	0	?	0	1	*Palaeobalistum orbiculatum*	?	0	2	1	1	1	1	0	2	0	3	1	1	?	0
?	?	?	?	?	?	0	0	?	?	?	?	?	?	0	*Paramesturus*	?	0	?	?	?	?	?	?	?	0	?	?	?	1	0
?	4	6	2	2	2	0	0	0	0	1	0	0	0	1	*Paranursallia gutturosa*	2	0	2	1	?	?	?	?	3	0	?	?	1	2	0
2	0	3	2	2	3	0	0	0	0	1	0	0	0	1	*Proscinetes*	2	0	2	2	0	0	0	0	2	1	2	2	1	2	0
4	0	5	2	3	3	0	0	0	0	[23]	0	0	0	1	*Pycnodus*	2	1	4	1	0	0	0	0	4	1	3	2	1	2	0
2	2	3	2	3	3	0	0	0	0	2	0	0	0	1	*Stemmatodus*	1	0	3	0	3	2	2	2	4	0	2	1	2	2	0
2	2	?	2	2	3	0	0	0	0	?	0	0	0	1	*Stenamara*	1	0	3	0	2	1	2	2	2	0	?	?	?	2	0
3	0	3	2	2	3	0	0	0	0	1	0	0	0	1	*Tepexichthys*	1	0	2	0	0	0	0	0	3	0	[02]	1	1	2	0
2	2	2	2	2	3	0	0	0	0	?	0	?	0	1	*Turbomesodon bernissartensis*	?	0	?	?	3	?	1	2	3	0	1	1	1	2	0

2	2	2	2	2	3	0	0	0	0	2	0	0	0	1	*T. praeclarus*	1	0	2	1	3	2	1	2	3	0	2	1	2	2	0
3	2	5	2	2	3	0	0	0	0	1	0	0	0	1	*T. relegans*	1	0	2	[12]	3	2	1	2	2	0	1	1	2	2	0
?	3	2	2	3	3	0	0	0	?	[13]	0	0	0	1	*Scalacurvichthys naishii* nov. gen., n. sp.	2	0	4	?	0	0	0	0	4	0	1	2	1	2	0

**101**	**102**	**103**	**104**	**105**
0	0	0	0	0
?	?	?	?	?
?	0	3	0	0
1	1	1	0	0
5	1	1	0	0
?	?	?	?	?
2	?	?	?	?
?	?	?	?	?
3	1	1	0	0
1	1	1	0	0
?	?	?	?	0
2	1	1	0	0
2	1	1	0	0
0	0	0	0	0
?	?	?	?	?
5	?	?	?	0
?	?	?	?	?
4	?	?	1	0
5	3	3	0	0
5	2	3	2	1
?	?	?	?	?
?	?	?	?	?
5	?	?	?	0
5	2	2	0	0
5	3	4	1	0
5	3	5	0	0
4	3	4	0	0
5	3	3	0	0
3	?	?	?	?

3	2	4	0	0
3	3	4	0	0
6	3	4	0	0
